# Tumor Heterogeneity and Molecular Characteristics of Glioblastoma Revealed by Single-Cell RNA-Seq Data Analysis

**DOI:** 10.3390/genes13030428

**Published:** 2022-02-25

**Authors:** Dhanusha Yesudhas, S. Akila Parvathy Dharshini, Y-h. Taguchi, M. Michael Gromiha

**Affiliations:** 1 Department of Biotechnology, Bhupat and Jyoti Mehta School of Biosciences, Indian Institute of Technology Madras, Chennai 600036, India; dhanusha2504@gmail.com (D.Y.); akilabioinfo@gmail.com (S.A.P.D.); 2 Department of Physics, Chuo University, Bunkyo-ku, Tokyo 112-8551, Japan; tag@granular.com

**Keywords:** glioblastoma, transcriptome analysis, tumor heterogeneity, biomarkers, network

## Abstract

Glioblastoma multiforme (GBM) is the most common infiltrating lethal tumor of the brain. Tumor heterogeneity and the precise characterization of GBM remain challenging, and the disease-specific and effective biomarkers are not available at present. To understand GBM heterogeneity and the disease prognosis mechanism, we carried out a single-cell transcriptome data analysis of 3389 cells from four primary IDH-WT (isocitrate dehydrogenase wild type) glioblastoma patients and compared the characteristic features of the tumor and periphery cells. We observed that the marker gene expression profiles of different cell types and the copy number variations (CNVs) are heterogeneous in the GBM samples. Further, we have identified 94 differentially expressed genes (DEGs) between tumor and periphery cells. We constructed a tissue-specific co-expression network and protein–protein interaction network for the DEGs and identified several hub genes, including *CX3CR1, GAPDH, FN1, PDGFRA, HTRA1, ANXA2 THBS1, GFAP, PTN, TNC*, and *VIM*. The DEGs were significantly enriched with proliferation and migration pathways related to glioblastoma. Additionally, we were able to identify the differentiation state of microglia and changes in the transcriptome in the presence of glioblastoma that might support tumor growth. This study provides insights into GBM heterogeneity and suggests novel potential disease-specific biomarkers which could help to identify the therapeutic targets in GBM.

## 1. Introduction

Glioblastoma multiforme (GBM) is a highly heterogeneous tumor, with diverse co-existing cell types that include tumor cells, endothelial cells, fibroblasts, and different cell types from the immune system [1,2]. Recently, it has been shown that the GBM subtypes can co-exist in different regions and cells within the same tumor [3]. Variability is found across tumor tissues, at different stages, and in different gender and age proportions. The invasive and metastatic ability of the tumor cells contributes to its high heterogeneity. The cells from the same tumor tissue can also have different mutations, which results in different phenotypic and epigenetic changes [4]. For example, the genes *SETD2, PTEN*, and *KDM5C* encountered multiple distinct and spatially separated inactivating mutations within a single tumor, and caused phenotypic evolution [5]. Similarly, Liu et al. [6] reported that the hypermethylation phenotype in the IDH1 mutant is involved in silencing of the α-KG-dependent DNA-modifying enzyme (Tet methylcytosine dioxygenase 2 (TET2)) and eventually increased the tumor formation. Tumor heterogeneity in patients and the characterization of its invasive nature remains a significant challenge for research and targeted therapeutic approaches [3,7]

Glioblastic cells affect stromal cells as well as the central nervous system (CNS) immune cells, including microglia, astrocytes, oligodendrocytes, neurons, and monocytes [8]. Maas et al. [9] reported that glioblastoma tumor cells communicate with microglial cells by releasing extracellular vesicles and these cells hijack the immune system. Similarly, cancer cells use tunneling nanotubes (TNT) as an efficient cell-to-cell communication system to adapt the microenvironment, which is also responsible for the invasive nature of the GBM tumors [10]. Neftel et al. [11] classified glioblastomas based on the intra and inter-tumoral cell state and genetic diversity of glioblastomas by comparing pediatric and adult glioblastomas. Macrophages, monocytes, and microglial cells are reported to be important in glioblastoma tumor growth, and glioblastoma invasion has been reduced with the depletion of these cells [12]. However, the exact pathways involved in tumor supportive process have not been characterized.

Although traditional bulk RNA-seq approaches have helped to identify key genes and pathways that drive GBM cells [13,14,15,16], they provide limited insights into the tumor heterogeneity and molecular mechanisms underlying GBM invasion. Darmanis et al. [17] reported the nature of infiltrating GBM cells and characterized neoplastic and non-neoplastic cells. However, other cell types, such as neural, glial, immune, and vascular cells, and the dynamic transition state of GBM cells remain unexplored. Hence, a better understanding of GBM heterogeneity between tumor and periphery cells and the molecular mechanism behind the transition of distinct cell types are necessary for further investigations.

In this work, we carried out a single-cell transcriptome data analysis of 3389 cells from four primary IDH-WT glioblastoma patients and provided a detailed analysis of the heterogeneity between the tumor and periphery cells. Further, we have carried out transcriptome studies based on the hg38 genome and with recent scRNA-seq analysis pipelines for preprocessing and downstream analysis. From copy number variation (CNV) analysis, distinct amplifications or deletions in different chromosomes have been observed for the patient samples. The novel and potent cell type-specific differentially expressed genes (DEGs) screened from different approaches, their functional enrichment and the co-expression networks, which have not previously been reported, strengthen our analysis regarding the disease-specific target genes. The potential DEGs are enriched with glial cell differentiation and mononuclear cell migration. Our study also explains the interplay between immune microglial cells and neoplastic cells and reveals the transition state of microglial cells.

## 2. Method

### 2.1. Data Collection and Quality Check

Darmanis et al. [17] reported high-depth single-cell RNA sequencing for a cohort of four primary GBM patients (IDH1-negative, grade IV GBMs confirmed via pathological examination). Two separate tissue samples were collected from each patient (one originating from the tumor core and another from the peritumoral space immediately adjacent to the tumor core, also termed the periphery). The details of the dataset are provided in Supplementary Table S1. Out of 3589 cells, 2343 cells are from tumor cores and 1246 cells are from the peripheral region [17].

**Figure 1:**
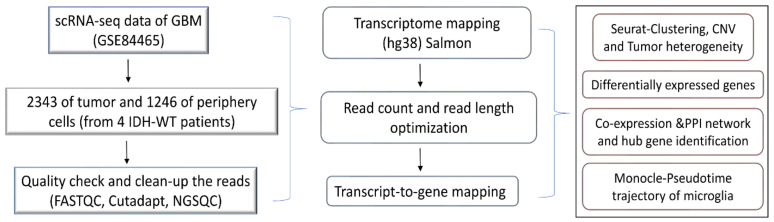
Schematic diagram for the study design.

The scRNA-seq raw reads were analyzed using FASTQC for a quality check and the short reads (less than 30) were discarded. The adapters were removed using cutadapt [18]. The processed reads were mapped against the hg38 human transcriptome using quasi-mapper “SALMON” (version 1.1.1) [19]. The transcripts were quantified and obtained as transcripts per million (TPM) values. The workflow of the present work is illustrated in Figure 1.

### 2.2. Dataset Preprocessing by Seurat

The TPM-based count matrix obtained from SALMON was taken as an input for the cell preprocessing, which was performed using the Seurat package [20]. We excluded the low-quality cells based on the following quality measures: (i) the genes showed expression in at least three cells and (ii) the minimum length of the RNA read should be 200 bps (Supplementary Figure S1). Mitochondrial genes were removed from the dataset as they represent contamination in the sequencing technique.

### 2.3. Dimensionality Reduction and Cluster Identification

The dimensionality of the dataset was determined by principal component analysis (PCA), and the number of PCs which captured the highest variance was obtained from the elbow plot (Supplementary Figure S1). Furthermore, the dataset was reduced to two dimensions with UMAP (Uniform Manifold Approximation and Projection). Clustering the groups of similar cells was performed with the FindClusters function based on the KNN algorithm. This is a graph-based clustering algorithm with edges drawn between cells with similar gene expression patterns. The “resolution” argument will set the “granularity” of the downstream clustering, which will be needed to be optimized for the experiments. In addition, we computed the average expression and dispersion of each gene using the module “FindVariableGenes” in Seurat and selected the top 1000 over-dispersed genes (outliers) for our study.

### 2.4. Determination of Copy Number Variations (CNVs)

InferCNV was used to explore the tumor single-cell RNA-seq data to identify large-scale chromosomal CNVs [21]. All genes were ranked by their chromosomal location, and the copy number of each gene was calculated as the sliding average of log2-transformed TPM values with a window of 100 flanking genes within each chromosome, which was then centered across all cells. Furthermore, we performed hierarchical clustering and removed the nontumor cells, which showed a few CNVs, similar to the normal cells. A hidden Markov model was used to predict the CNV states, and the gene location data were obtained from the Biomart database.

### 2.5. Differential Gene Expression and Functional Annotation

Tximport was used to import the transcript level estimates (TPM values) from SALMON and summarize them as gene abundance. Subsequently, DESeq2 software [22] was used to identify the genes, which were differentially expressed in tumor core and periphery cells. The genes with log2FC > 1 and log2FC < 1 with adj. *p*-value < 0.05 were considered as significant differentially expressed genes. Benjamini and Hochberg’s method was used to get the adj. *p*-value.

“FindMarkers” and “FindAllMarkers” from Seurat were used to identify the DEGs, and a gene expression level with log2FC > 1 and an adj. *p*-value < 0.05 were used as filtering criteria. The commonly observed DEGs from DESeq2 and Seurat were filtered for further analysis. The ontology and functional annotations of DEGs were analyzed using ClueGO from Cytoscape.

### 2.6. Network Analysis

The significantly differentially expressed genes were analyzed using the STRING and Humanbase databases in order to analyze the interacting partners [23]. The protein–protein interaction network was generated from STRING, and the tissue (human cortex) specific co-expression network was generated from Humanbase. Cytoscape plugin MCODE was used to find the cluster and hub genes.

### 2.7. Monocle Pseudotime Trajectory Reconstruction and Analysis

Single-cell pseudotime trajectories were constructed with MONOCLE 2. This method uses the reverse graph embedding (a machine learning technique) method to reduce the given high-dimension expression profile to low-dimensional space [24]. Single-cell data points were projected onto this low-dimensional space and ordered into a trajectory with branch points. This method tests whether differences in gene expression are associated with particular branching events on the trajectory.

## 3. Results

### 3.1. Cell Clusters and Tumor Heterogeneity in GBM

**Figure 2:**
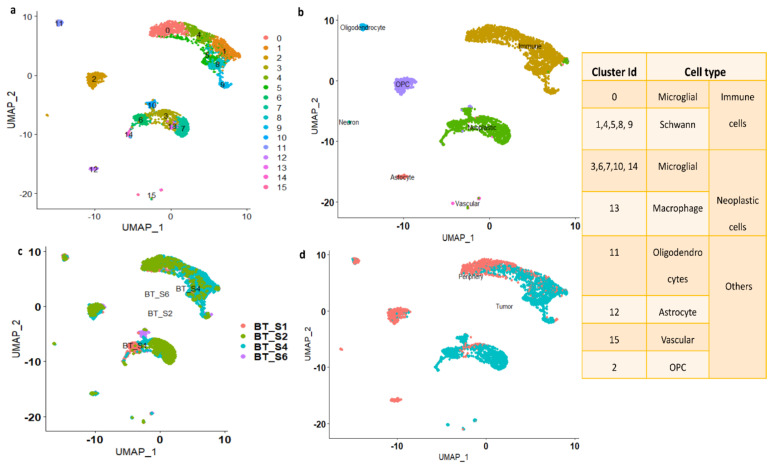
(**a**) UMAP plot showing the distribution of cells with cluster ID. (**b**) UMAP clusters are labeled based on major cell types. (**c**) Clustering of the cells based on patient-ID. (**d**) Clustering of the cells based on tissue. The table displays the cluster ID matches with cell type.

A schematic diagram of the study design and the principal findings are shown in Figure 1. A total of 2343 tumor cells and 1246 periphery cells from four IDH-WT patients were adapted for this study (Supplementary Table S1). After stringent quality control and normalization, we analyzed these 3389 cells with 42,970 genes. In order to identify and characterize GBM cellular heterogeneity, we clustered the similar cells using UMAP (see Materials and Method). UMAP computed a total of 16 clusters for the 3389 human GBM cells (Figure 2). PCA variance analysis captured the highly variable genes across the PCs (Supplementary Figure S1b) and the top 1000 cell-to-cell variable genes are reported in Supplementary File S1. The positive and negative correlation of these variable genes along with their PCs are represented in Supplementary Figure S1c.

#### 3.1.1. Cell Type Identifications of the Clusters

Based on metadata and marker gene identification, the UMAP identified clusters were grouped into seven major cell types (astrocyte, oligodendrocyte, vascular, OPC, neuron, immune, and neoplastic), as shown in Figure 2b. From each cluster, genes showing significant expression changes (log2FC > 1 and adj. *p*-value < 0.05) were filtered out and annotated with ScCATCH and PanglaoDB databases and subsequently labeled as potent markers for the cell types.

Clustering based on cluster ID: As seen from Figure 2b, the immune cluster is the largest cluster comprising 0, 1, 4, 5, 8, and 9 subclusters. Cluster 0 contains 540 cells and is annotated as microglial cells; clusters 1, 4, 5, 8, and 9, containing 1290 cells, are annotated as Schwann cells (a type of glial cell). The second largest cluster is the neoplastic cluster, comprising 3, 6, 7, 10, 13, and 14 subclusters. Clusters 3, 6, 7, 10, and 14, containing 945 cells, are annotated as microglial cells, and subcluster 13 contains 49 cells and is annotated as macrophages. Clusters 2, 11, 12, and 15 are annotated as OPCs, oligodendrocytes, astrocytes, and vascular cells, respectively. The number of cells in each cluster is represented in Supplementary Figure S1e.

Clustering based on patient ID: Figure 2c shows the clusters which are grouped based on patient ID. The patient BT_S2 and BT_S4 samples are highly crowded with immune and neoplastic cells. However, the patient ID BT_S1 and BT_S6 samples are mainly occupied with neoplastic cells (Figure 2c).

Clustering based on tissue: The clusters are grouped based on tissue characteristics and the data are shown in Figure 2d. The neoplastic cells are mainly from tumor core cells. However, the immune clusters contain both tumor and periphery core cells.

#### 3.1.2. Marker Genes in Each Cluster

**Figure 3:**
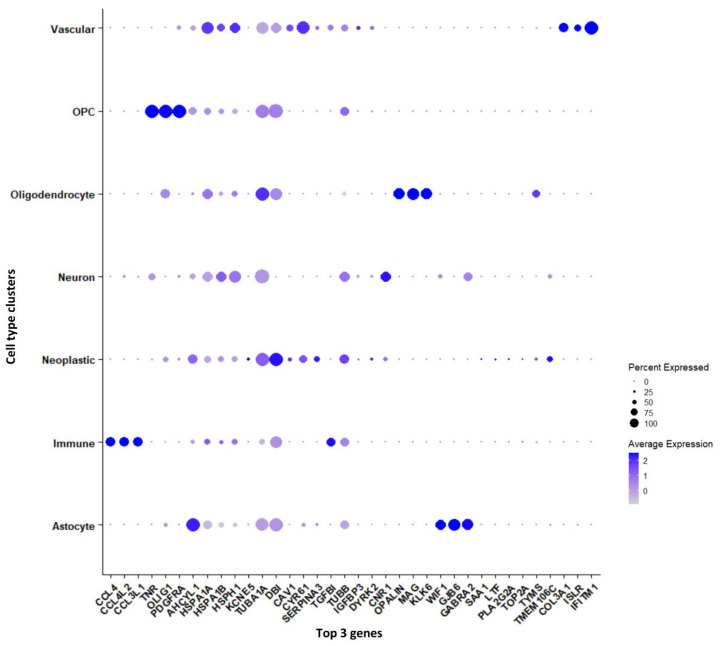
Top three marker genes and their expression unique to each cluster represented in a dot plot.

Genes that are significantly expressed in each cell type (astrocytes, immune cells, neurons, neoplastic cells, OPCs, oligodendrocytes, and vascular cells) have been reported in Supplementary File S1. Furthermore, we have reported the genes, which are differentially expressed between tumor and periphery cells and unique to each cluster, shown as a dot plot in Figure 3. The cytokine genes *CCL4, CCL4L2,* and *CCL3L1* are down-regulated in tumors and are up-regulated in periphery cells, and these genes are found to be markers for the immune cell cluster. The genes *TNR, OLIG1,* and *PDGFRA* are specific to the OPC cluster and are differentially expressed in tumor and periphery cells. These genes are up-regulated in tumors and down-regulated in periphery cells. It has been reported that co-expression of *EGFR* and *PDGFRA* is a driver event early in GBM [25,26]. The heat-shock genes *HSPA1A* and *HSPA1B* show down-regulation in tumor and up-regulation in the periphery, and act as marker genes for neuron clusters. *TUBA1A, DBI,* and *TUBB* are up-regulated in tumor cells and down-regulated in periphery cells, and these are marker genes for the neoplastic cluster. The tubulin proteins *TUBA1A* and *TUBB* and their heterogeneity has been associated with GBM, whereas *DBI* maintains high proliferation rates, promoting tumor growth [27]. The oligodendrocyte marker genes *OPALIN, MAG,* and *KLK6* are up-regulated in tumor cells and down-regulated in the periphery. The highly variable genes *MAG* (Supplementary Figure S1b) and *OPALIN* encode glycoproteins involved in myelinating oligodendrocytes. The genes *COL3A1, ISLR*, and *IFITM1* are specific for vascular cells and show up-regulation in the tumor and down-regulation in the periphery. The genes *COL3A1* and *IFITM1* are involved in cell migration, and *ISLR* is the contemporary gene identified from this current study.

### 3.2. Differentially Expressed Genes (DEGs) between Tumor and Periphery Cells

Considering the DEGs from each cluster, we were interested to infer the differentially expressed genes in the tumor and periphery cell populations, so we used DESeq2 and Seurat to analyze the differentially expressed genes between the tumor and periphery cells. We compared the results from DESeq2 and Seurat and the consensus results are discussed below.

**Figure 4:**
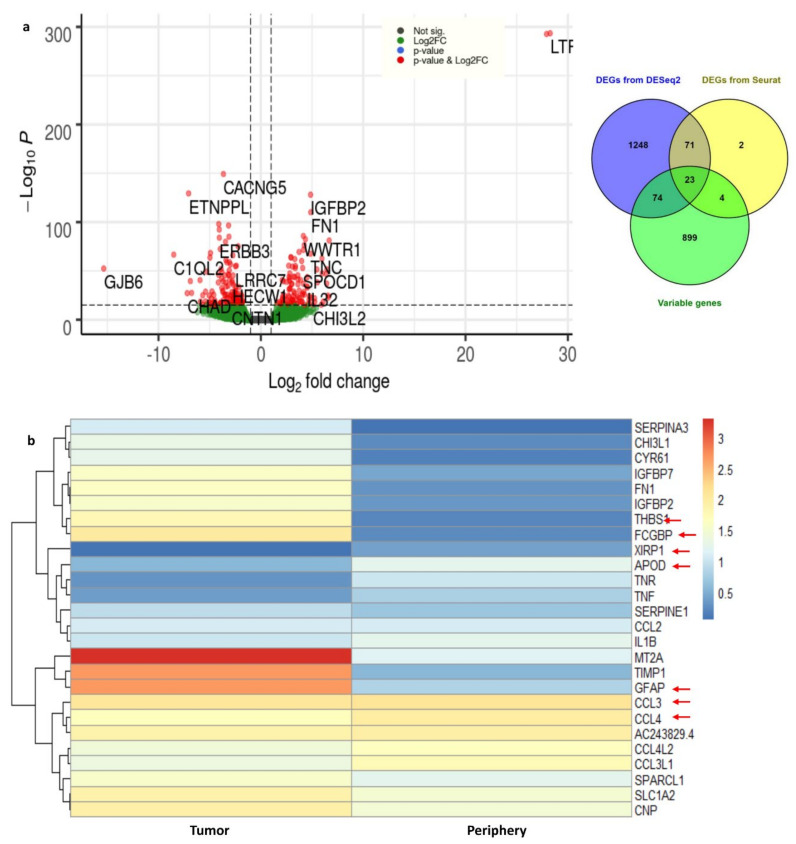
(**a**) Volcano plot for the differentially expressed genes from DESeq2. The up- and down-regulated genes with the cut-off of log2FC > 1 and with adj. *p*-value < 0.05 are shown in the figure. (**b**) The heat map representation for the important DEGs and their expression rates in tumor and periphery core tissues.

Using DESeq2, the DEGs from tumor and periphery cells were obtained. With 3389 cells, a nonzero read count of 21,250 was obtained. We have applied the filters adj. *p*-value < 0.05, log2FC > 1 for up- and log2FC < 1 for down-regulated genes. We obtained 2.1% up-regulated (454 genes) and 3.2% down-regulated genes (680 genes). The outliers and the low counts had been removed. Figure 4 shows the clear separation of up- and down-regulated genes, with significant genes marked in red.

DEGs from Seurat were filtered with log2FC > 1 and adj. *p*-value < 0.05 and 100 genes (33 up-regulated and 67 down-regulated) were obtained. The complete list of differentially expressed (up- and down-regulated) genes from DESeq2 and Seurat are presented in Supplementary Table S2. We considered the common DEGs from DESeq2 and Seurat, resulting in 94 significant differentially expressed genes. Among them, 22 DEGs are novel in the GBMs found in this study. Furthermore, *DHRS9, IPCEF1, TNR, MEGF11, EDIL3, PDZD2, ATP1A2, PDGFRA,* and *MEG3* are novel down-regulated genes that are involved in focal adhesion, cell adhesion to the extracellular matrix (ECM), thereby allowing the cells to crawl during migration. Similarly, the genes *CHI3L1, FN1, IGFBP2, TNC*, *FCGBP, CYR61, F13A1, ANXA2, AC006064.4*, and *MIF-AS1* are novel up-regulated genes which are involved in ECM–receptor interactions and collagen cross-linking. Integrins interact with ECM components, such as collagen, brevican, tenascin-C, fibronectin, and thrombospondin, which leads to the adhesion and migration of glioma cells [28].

These 94 genes were again checked for overlap with highly variable genes which were obtained from Seurat (top 1000 genes). We ended up with 23 common genes (Supplementary Table S3); the expression of these important genes in tumor and periphery conditions are shown in Figure 4b. The heatmap shows the differential expression of these key genes in tumor and periphery tissues. Mainly, the cytokine genes (*CCL3, CCL4, CCL3L1,* and *CCL4L2*) show moderate expression in tumor core cells and show less expression in periphery cells, which correlates with the results from Darmanis et al. [17]. *MT2A, TIMP1*, and *GFAP* are more highly expressed in tumor cells and are down-regulated in the periphery. The change in the expression level of the novel gene *AC243829.4* is not distinct; however, this lncRNA function is not reported. Similarly, the genes *FCGBP, THBS1, IGFBP2, IGFBP7,* and *FN1* are down-regulated in periphery cells and up-regulated in a disease condition. These 23 common genes are mainly involved in ECM–receptor interactions and epithelial–mesenchymal transitions (EMTs). EMT is a dynamic process of converting epithelial cells to mesenchymal phenotypes. Hypoxia, cytokines, growth factors secreted from the tumor environment, and treatment with anticancer drugs act as inducers for the EMT process [29]. Eventually, EMTs can create tumor cells with stem cell properties that are more aggressive and can increase their metastatic activity [30].

### 3.3. Tumor Heterogeneity with CNV Profiles

Genomic CNVs are commonly associated with tumor initiation and progression [31]. Thus, we attempted to infer large-scale copy-number alterations for each cell by averaging its relative expression levels over large genomic regions. This allowed us to suppress the individual gene-specific expression patterns and emphasize the signal of large-scale CNVs. InferCNV package was used to calculate CNV vectors for each cell and then clustered cells based on their respective profile CNV vectors (the details are given in the section on Method).

**Figure 5:**
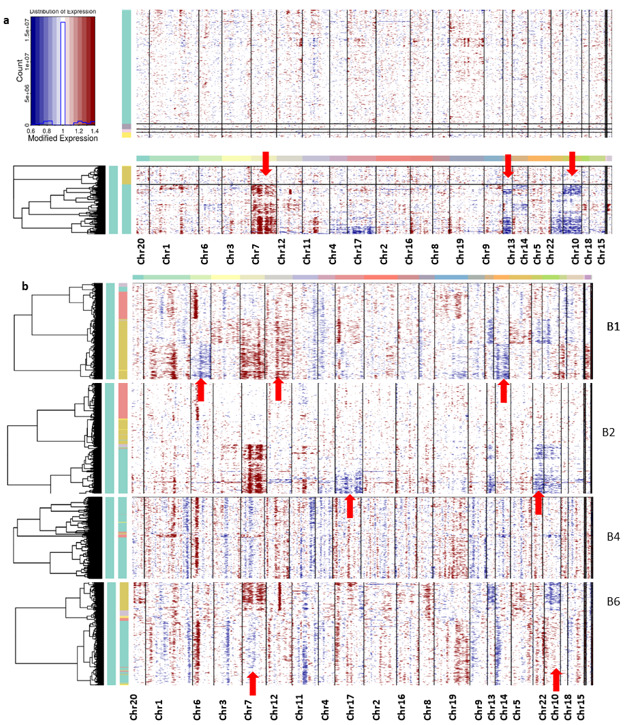
(**a**) Inferred chromosomal CNV profiles of complete cells based on average relative expression in a window of 100 genes. Oligodendrocytes that lack CNVs are shown as a reference group at the top. (**b**) The CNV profile of each patient’s sample cells. Red indicates amplifications and blue indicates deletions.

Overall, the non-neoplastic cells did not show any chromosomal abnormalities, only the neoplastic cells from all the patients having aberrations in their chromosomes (Figure 5). The CNV profiles revealed the coherent chromosomal aberration in each tumor cell. From the CNV profile, the gain of chromosome 7 expression and the loss of chromosomes 10 and 13 were constantly observed from the cells from all patient samples.

We mapped the DEGs which are located at chromosomes 7 and 10, which exhibited the copy number alternations in these chromosomes. Further, we filtered the genes based on transcript levels (gene expression) and observed that 10 potential DEGs are involved in the CNV of chromosome 7. The copy number gain of chromosome 7 is associated with the up-regulation of genes *ANLN, SRI, PON2, ITGB2, PTN, CAV1, PTPRZ1, MEST, CALD1, RAPGEF5,* and *NDUFA4*, which are mainly involved in epithelial–mesenchymal transitions. The down-regulated genes *NPTX2, PTPRZ1, EGFR*, and *COL1A2* also showed associations with the copy number gain of chromosome 7 and are involved in epithelial cell signaling. *EGFR* is the most frequently amplified oncogene in astrocytic tumors; expression of genes *SRI, NPTX2, MEST, RARRES2*, and *SEPTIN7* in association with GBM is reported in this study.

Similarly, the genes *SCD, EGR2, HTRA1, PIP4K2A, PHYHIPL, PSAP,* and *LINC00844* showed significant down-regulation with the loss of chromosome 10. These genes are mainly involved in Schwann cell differentiation and among them *SCD, OPALIN,* and *PHYHIPL* are novel genes. Wang et al. [32] reported that the Linc RNA (*LINC00844*) is associated with glioblastoma prognosis, cell proliferation, invasion, cell cycle, and metastasis [32]. However, genes *VIM* and *PPA1* showed up-regulation with the loss of chromosome 10. The up-regulation of *VIM* protein and its role in the formation of lamellipodia and invadopodia during cell invasion and migration have already been reported [33]. Prosaposin (*PSAP*) is highly expressed and secreted in gliomas and can promote glioma invasion and epithelial–mesenchymal transitions. Significant expression changes of these genes might also be the reason for the CNV change. A list of genes responsible for the chromosome alteration along with its expression changes is tabulated in Table 1.

Interpatient heterogeneity is also studied from this CNV profile; the unique CNV abnormalities of each patient sample cell are described below. The CNV profile from each patient sample has been captured for deeper understanding (Figure 5b). It can be seen in the patient B1 sample that the neoplastic cells show the major changes, especially in the loss of chromosome 12 and the gain of chromosomes 6 and 14, whereas B2 samples show a gain of chromosomes 17 and 22. However, samples from patient B4 did not show significant changes in any chromosomes. Neoplastic cells from patient B6 samples display the disorder copy number changes in chromosomes 7, 10, and 13 in the neoplastic cells. The DEGs are located on chromosomes 6, 7, 10, 12, 13, 17, and 22, and their expression change is tabulated in Table 1.

### 3.4. Pathway/Function Enrichment Analysis for DEGs

The significant 94 DEGs and their gene ontology terms were calculated using ClueGO analysis. Gene ontology (GO) functional enrichment analyses [34] were used to determine the potential molecular mechanisms employed by these 94 important genes (33 up-regulated and 61 down-regulated). The pathways in which these genes are involved are discussed below.

**Figure 6:**
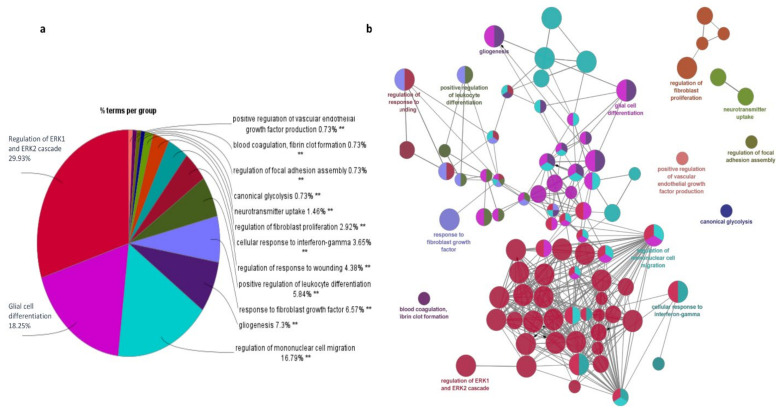
Functional annotations of the important DEGs (94 overlapping genes) obtained using ClueGO. (**a**) Go enrichment analysis; (**b**) Expression regulation network of DEGs.

ERK1 and ERK2 cascade: ClueGO analysis revealed that regulation of the ERK1 and ERK2 cascade is the major pathway experienced by the DEGs (Figure 6). Extracellular signal-regulated kinase plays a central role in transmitting extracellular signals to intracellular targets, including proliferation, differentiation, and survival. It has been reported that the ERK pathway is aberrantly activated in malignant gliomas [35]. In total, 16 genes actively participate in the ERK signaling cascade, of which 5 genes are up-regulated and 11 genes are down-regulated. The genes *CCL2, CCL3, CCL3L1, CCL4, CCN1, CD44, CHI3L1, CSF1R, DUSP6, FN1, IL1B, MIF, PDGFRA, PDZD2, TIMP1*, and *TNF* from our study are shown to be involved in the ERK1 and ERK2 pathway. Among them, *CD44, CHI3L1, FN1, MIF*, and *TIMP1* are up-regulated, whereas *CCL2, CCL3, CCL3L1, CCL4, DUSP6, CSF1R, IL1B, PDGFRA, PDZD2*, and *TNF* are down-regulated. *CHI3L1* binding to *CD44v3* activates ERK protein, *CD44* and *MIF* are required to activate the MEK–ERK–MAP kinase pathway [36]. The binding of *FN1* to integrins induces conformational changes and activates the recruitment of focal adhesion kinase (FAK) [37]. FAK signaling enhances the activation of metalloproteinase and stimulates the diffuse nature of cells through ECM. ERK cascade signaling is essential to the production of inflammatory cytokines, and this signaling pathway is hyperactive in malignant gliomas due to overexpression of *EGFR* and *PDGFR* [38].

Glial cell differentiation: The main function of glial cells is to provide support to neurons and maintain hemostasis. The DEGs identified from our study involved in glial cell differentiation are *CNP, DNER, EGR2, GFAP, GPR37L1, GSN, OLIG1, PTN, TNF,* and *VIM.* Among them, *GFAP, PTN*, and *VIM* are up-regulated and *CNP, DNER, EGR2, GPR37L1, GSN, OLIG1,* and *TNF* are down-regulated. The gene DNER is an epigenetically modified gene that enhances GBM progression. The genes *GFAP* and *VIM* are reported as glial cell markers, whereas *CNP* and *OLIG1* are novel genes from our study for glial cell differentiation. Generally, glial cells differentiate into neuron-rich and neuron-free regions which include astrocytes and oligodendrocytes. GFAP-positive radial glial cells transform into astrocytes, whereas *OLIG2* is involved in glial cell transformation into oligodendrocytes [39].

Mononuclear cell migration: Mononuclear cells are mainly monocytes and lymphocytes and these cells are critical components of the innate and adaptive immune system. The genes *ANXA1, APOD, C3AR1, CCL2, CCL3, CCL3L1, CCL4, CH25H, CSF1R, CX3CR1, THBS1*, and *TNF* are identified as important genes and are involved in mononuclear cell migration. We noticed that *ANXA1* and *CH25H*, which are involved in mononuclear cell migration, are reported here for the first time. The genes *ANXA1* and *THBS1* are up-regulated and *CCL2, CCL3, CCL3L1, CCL4, APOD, CX3CR1,* and *C3AR1* are down-regulated. The mononuclear cell migration pathway is associated with cell adhesion, leukocyte migration, chemotaxis, and inflammatory response. *CCL2, CCL4*, and *TNF* are mainly involved in all the pathways except cell adhesion, whereas *FN1* and *TNF* are involved in the cell adhesion pathway.

Fibroblast migration: Cytokines and growth factors, which are released as an inflammatory response, attract the fibroblast into the wound site, consequently starting the repair process. The genes *CCL2, CD44, EGR3, NR4A1, SULF2, THBS1,* and *TNC* are identified as potent genes for fibroblast migration. The genes *DE44, THBS1*, and *TNC* are up-regulated; *CCL2, EGR3, NR4A1*, and *SULF2* are down-regulated. Regulation of *NR4A1* and *TNC* expression in glioblastomas remains unreported; however, *TNC* is an ECM receptor protein that involves ECM receptor interactions. The PI3K–Akt–mTOR, FGF, and ERK signaling pathways are involved in fibroblast migration. *EGR3*, *CD44*, and *CCL2* are known to be involved in the ERK signaling cascade.

Active migration is in the nature of glioma cells; it is a dynamic process of interaction between tumor cells and their microenvironment. Further, tumor cell attachment to the ECM plays an important role in tumor progression. The common feature of all GBM types is that aberrant kinase signaling includes the PI3K–Akt, MAPK, and ERK1/2 signaling pathways. Hannen et al. [40] suggested that ERK1/2 signaling is activated by phosphorylation in GBM (mainly in the mesenchymal subtype). A hypoxic microenvironment is created due to signaling disruptions which will lead to the recruitment of macrophages and microglia and to the release of growth factors (EGR, TGF, HGF, PDGF, HIF, and IGF1). The potential growth factors will be the inducers for epithelial–mesenchymal transitions (EMT) and numerous proteases that increase invasiveness into the surrounding normal brain [41,42,43].

### 3.5. The Transition of Microglial Immune Cells to Neoplastic Cells: Pseudotime Analysis

The main function of most of the DEGs is related to glial cell differentiation. Cell migration is mainly governed by the differentiation of microglial cells present in the brain. Usually, for healthy people, microglial cells in the brain regulate tissue homeostasis by surveying their environment. Their functions include phagocytizing the synaptic elements, living/dying cells, and apoptotic cells. In GBM disease conditions, where microglia are in close interaction with neurons, astrocytes and oligodendrocytes can shift into different functional states, modifying GBM proliferation and morphology [44]. During the response to inflammation or tumor growth, these microglial cells alter their morphological appearances and sometimes retract their ameboid appearance. These phenotypic changes in the immune cells have been associated with patient prognosis, though the detailed mechanism/crosstalk between GBMs and microglia is poorly understood [45,46,47]. Chen et al. [45] reported that the transition state of microglia significantly altered disease prognosis. Glioblastomas recruit neighboring resident microglia through the secretion of various chemokines and cytokines. *CCL2* and *CCL3* are associated with monocyte and macrophage recruitment and may act as chemoattractants to attract other microglial cells, causing disease invasion [48,49]. Bachiller et al. [44] showed that aging causes changes in gene expression as well as in the occurrence of dystrophic microglia. These changes related to aging might have an impact on the progression of neurodegenerative disorders [44].

**Figure 7:**
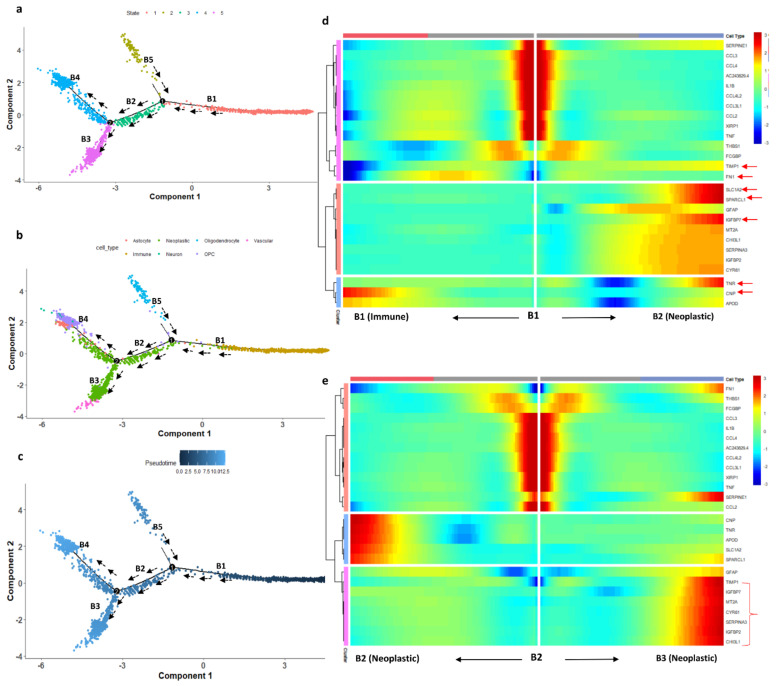
(**a**) DDR three-dimensional reduction of the cells computed using Monocle 2 contains five main branches; the cells took the path based on the pseudotime represented with the black arrow. (**b**) Trajectory states based on cell type. (**c**) Trajectory based on pseudotime. Dark blue represents the initial state (less differentiated) of the immune cells and light blue (more differentiated) represents the differentiated cell state (neoplastic cells). (**d**) Heatmap depicting the transition state of microglial marker genes in a branch-dependent manner for root points 1 and 2. Each row represents the dynamic expression of a gene. (**e**) Branch point 2 set as the root; the left arrow shows the root to the trajectory path 2; the right arrow shows the root to trajectory path 3.

We studied the differentiation state of microglial cells using pseudotime trajectory analysis. The immune cell landscape differs strongly between infiltrating and central regions of glioblastomas and changes from one cell type to another. Monocle 2 constructs the single-cell trajectories in pseudotime, which consist of two branch points (five branches and five states). The trajectory roots 1 and 2 (based on branch points 1 and 2) are more populated with immune and neoplastic cells, respectively (Figure 7). The trajectory starting state (state 1, dark blue) is crowded with immune cells. The highly differentiated cells (light blue) are populated with neoplastic cells (end-state 5). The transition paths from immune to neoplastic cells are represented with black arrows. The proposition of cells in each state also represents that the starting immune and neoplastic cell states have the highest proposition of cells (state 1 has 1829 cells and states 4 and 5 have 577 and 703 cells, respectively). The immune cells, especially those from cluster 0, are mainly microglial cells, which have migration potential, differentiating from states 1 to state 5 through state 3. To confirm this assumption, we used a heatmap to confirm the transition of microglial genes from immune cluster marker genes. The DEGs from the immune cluster which show significant expression changes are filtered out and used for the heatmap. The branch-dependent trajectory analysis of these microglial marker genes is shown in Figure 7b. As seen from Figure 7b, the marker genes *FN1, SLC1A2, SPARCL1, IGFBP7, MT2A, CYR61, CHI3L1,* and *TIMP1* have expression changes while traveling from root to branches B1 and B2, whereas *TNR*, *CNP*, and *APOD* show expression changes towards immune cells. Branches 2 and 3 are enriched with neoplastic cells. We set the root as branch point 2 and the genes show significant expression change while traveling towards B3 (neoplastic cells). The microglial immune cells are experiencing the transition state in pseudotime, which is observed with the heatmap analysis.

### 3.6. Protein–Protein Interaction Networks (PPI) and Tissue-Specific Co-Expression Networks for DEGs

#### 3.6.1. PPI Network in DEGs

**Figure 8:**
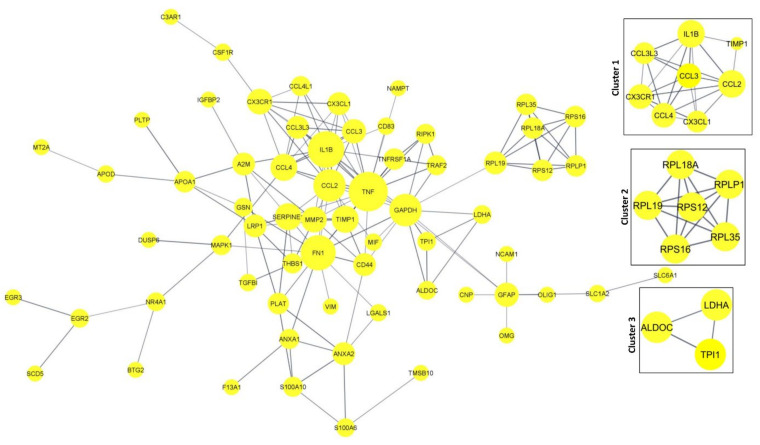
PPI network for the significant DEGs. Cluster and Hub gene screening from the network were performed using MCODE of Cytoscape.

The protein–protein interaction network and the tissue-specific gene co-expression network for the significant DEGs were obtained from the STRING and HumanBase databases, respectively. The 94 DEGs obtained from DESeq2 were provided as inputs to the STRING database for PPI networks with a confidence score of 0.8 (Figure 8). The network was obtained from the STRING database with 103 nodes and 149 edges (PPI enrichment *p*-value < 1.0 × 10^−16^).

The Cytoscape plugin MCODE was used for clustering the network and filtering out the hub genes. From MCODE we obtained cluster 1 with a score of 6.286 (eight DEGs), cluster 2 with a score of 6 (six DEGs), and cluster 3 with a score of 3.3 (three DEGs) (Figure 8). Network node sizes are represented based on the degrees of connectivity. The PPI network shows that *TNF, CCL2, IL1B, FN1, CCL4*, and *GAPDH* are the important genes having a greater number of interactions with the high degree of 6. All other genes except *GAPDH* are down-regulated. Based on the degrees of connectivity and the cluster analysis, *RPL19, CX3CR1, LDHA, TPI1, RIPK1, TRAF2, GFAP, APOA1, PLAT, TNF, CCL2, IL1B, FN1, CCL4, GAPDH*, and *ALDOC* are termed hub genes for the interactions. These hub genes are mainly involved in the vitamin B12 metabolism pathway. The important biological function of vitamin B12 is to accomplish DNA synthesis, which is necessary for cell division. All the living cells require vitamin B12 for survival and it strongly promotes glioblastoma cell proliferation; therefore, B12 deficiency is not favorable to GBM prognosis [50].

#### 3.6.2. Tissue-Specific Co-Expression Network of DEGs

**Figure 9:**
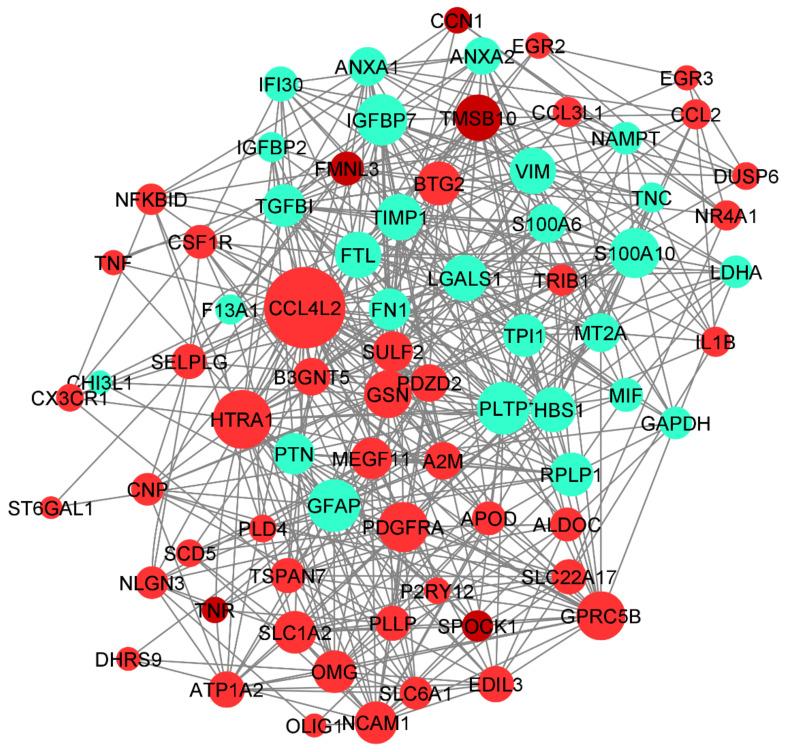
Co-expression network of DEGs constructed using Humanbase and visualized with Cytoscape. Proteins are represented with color nodes; the size of the nodes represents the degree parameter. The networks for up- and down-regulated genes are represented in red and cyan, respectively.

The human base tissue-specific network displays the co-expression network specific to the 94 DEGs. The reference network was obtained from human brain cortex tissue; the DEGs were mapped against the reference network and our co-expression network was constructed (Figure 9). The genes *CCL4L2, HTRA1, GFAP, PLTP, PDGFRA,* and *GSN* constitute the central part of the network with high closeness centrality (~0.6) and high-degree parameters (49). *GFAP* protein-expressing neural stem cells (NSCs) are responsible for the activation of PDGF receptors and their stimulation, which eventually form the glioma-like mass [51]. *PDGFRA* is also responsible for proinflammatory cytokines, including *CCL4L2*. We have identified the genes co-expressed together: gene *CCL4L2* is co-expressed with *PLTP, SELPLG, ANXA2, RPLP1*, and *P2RY12*. *CCL4L2, SELPLG*, and *P2RY12* are down-regulated, whereas *PLTP, RPLP1*, and *ANXA2* are up-regulated. The genes *SULF2, PTN, FTL, TPI1, LDHA, THBS1,* and *TGFBI* are co-expressed together. *CCL3L1* interaction with *ATP1A2* has the highest centrality; *HTRA1* interaction with *B3GNT5, GFAP, PLTP,* and *GSN* is shown to form the highest interaction network (Figure 9).

Increased expression of *B3GNT5* strongly correlated with the progression of breast cancer, lung cancer, and ovarian cancer. However, *B3GNT5* is identified as a novel gene for GBM progression. Copy number alteration of *HTRA1* is strongly associated with poor prognosis of GBM; however, there has been no clear study of the expression level of *HTRA1*. Therefore, the interaction of *HTRA1* with *B3GNT5, GFAP, PLTP*, and *GSN* could be a novel identification in the GBM disease mechanism.

## 4. Discussion

In this study, we utilized scRNA-data to explore the molecular cascade of GBM disease progression. Initially, we performed a single-cell transcriptome analysis to characterize tumor heterogeneity and the molecular mechanism of GBM invasion. It has been reported that GBM comprises morphologically and phenotypically diverse cells and cell types [4]. We have identified 7 major clusters and 16 subclusters from the population of 3389 cells. The functional annotation of the cluster-specific marker genes displays distinct gene ontology, representing the heterogeneity of the cells. The CNV profile explains the intra-patient tumor heterogeneity and the data shown in Figure 5 demonstrate the distinct large-scale chromosomal variation across individual patient samples. The gain of chromosome 7 and the loss of chromosomes 10 and 13 are common to all the patient sample cells, and the chromosomal aberrations are mainly caused by neoplastic cells.

According to experimental reports, the gain of chromosome 7 has been associated with the simultaneous loss of chromosome 10 and suggests that *EGFR* amplification and the deletion mutation of *PTEN* is associated with the abnormalities of chromosomes 7 and 10, respectively [52]. Along with chromosomes 7 and 10, abnormalities in chromosomes 12, 13, and 6 have also been reported for patients with human gliomas, Alzheimer’s disease, and Parkinson’s disease [53]. Transcriptional downregulation of the *NDRG2* gene on chromosome 14 has been observed in primary glioblastoma patients [54]. All the above-reported genes are also observed to be the same in our study. Loss of chromosomes 17 and 22 have been reported in gliomas; however, we have observed a gain in chromosomal abnormalities [55,56]. This might be because, except for the *SOX9* oncogene, the chemokine genes (*CCL2, CCL3, CCL4,* and *CCL4L2*) of chromosome 17 are up-regulated. Apart from these reported genes, other novel DEGs contributing to chromosomal abnormalities are tabulated in Table 1. However, further experimental results and analysis are needed for these genes.

We have identified the DEGs from tumor and periphery cells, suggesting the functionally important genes. Darmanis et al. [17] identified 30 potent differentially expressed genes across neoplastic and non-neoplastic cells, and among them *CA2, GAP43, PMP2, CRYAB, SOX9, EGFR*, and *ATP1A2* are reported to be DEGs between tumor and periphery cells in our analysis. The filtered DEGs are further used for the PPI and co-expression network construction. The DEGs *DHRS9, IPCEF1, TNR, MEGF11, EDIL3, PDZD2, PDGFRA, SPOCK1, CHI3L1, FN1, IGFBP2, TNC, FCGBP, CYR61, F13A1, ANXA2 NCAM1, RPL19, SLC1A12, CNP, MT2A, CHI3L1, POSTIN, LTF, MPDZ, CPZ, LRRC32, CTNNA3, LRFN5*, and *SLc22A17* reported in our study are not explored by many other studies related to GBM. The novel genes identified in our study have been tabulated in Table 2. The main functions of the DEGs are mostly involved in the ERK1/2 signaling cascade, glial cell differentiation, and mononuclear migration. These main pathways are interlinked with ECM cell adhesion and adhesion to surrounding cells, and the ECM is important for GBM cancer cell maintenance. The DEGs are involved in cell adhesion and ECM receptor interactions. It is evident that the above-mentioned potent DEGs and the novel DEGs play an important role in GBM progression. Targeting these DEGs and their pathway might help for therapeutic applications.

We have also explored glial cell differentiation by identifying the microglial cell transition state by single-cell trajectory reconstruction. The pseudotime analysis revealed that the microglial cells differentiate into neoplastic cells. The trajectory analysis identified the pseudotime starting state as immune cells and the ending state as neoplastic cells. We determined the path along which cells traveled from root to branch, representing the transition of the cells (Figure 7). Some of the marker genes from microglial cells, such as *FN1, SLC1A2, SPARCL1, IGFBP7, MT2A, CYR61, CHI3L1*, and *TIMP1*, showed a gradual increase in expression while they traveled from root to branch. A glioblastoma takes control over the microglial immune cells of periphery tissue by various kinds of cytokine and chemokine signaling [9]. The invasive microglial cells from periphery cells are a possible target for the GBM disease breakthrough.

### Comparison with Other Related Works

Several investigations have been carried out to understand the tumor heterogeneity of glioblastomas using RNA-seq analysis [57]. Patel et al. [4] showed that the glioblastoma subtype classifiers are variably expressed across individual cells and discussed the intra-tumoral heterogeneity within a tumor. Neftel et al. [11] compared the scRNA-seq of 20 adults and 8 pediatric glioblastomas (24,131 cells in total) and identified four main cellular states. Darmanis et al. [17] reported the characterization of neoplastic and non-neoplastic cells and determined that the neoplastic cells share common characteristics regardless of the patient of origin. However, GBM heterogeneity across tumor and periphery cells and the molecular mechanism underlying the transition of distinct cell types are not completely explored.

We have identified cell type-specific DEGs and DEGs between tumor and periphery cells to characterize tumor and periphery cells. The filtered cell type-specific DEGs are used for hub gene identification using PPI and co-expression network analysis, and 51 novel genes were identified in our study (Table 2). Among the novel differentially expressed genes, *IPCEF1, F13A1, TPI1, B3GNT5, ATP1A2, FN1, PDGFRA*, and *GSN* are found to be hub genes, and we suggest that these genes could be potential therapeutic targets in GBM disease prognosis. In addition, we compared the significantly expressed genes identified in our analysis with other experimental studies reported in the literature [11,17,58,59,60,61] and the results are presented in Supplementary File S2. We observed that 422 genes can be matched with other studies.

We have also explored the transition state of microglial immune cells during the invasion process with pseudotime analysis. The trajectory path starts with immune cells and ends with neoplastic cells. The differentiation state of immune microglial genes (*FN1, SLC1A2, SPARCL1, IGFBP7, MT2A, CYR61, CHI3L1*, and *TIMP1*) showed the expression changes while traveling from the root to the end state (Figure 7). In addition, we were able to capture the patient-wise chromosome alterations in chromosomes 13, 14, 17, and 22, and the genes responsible for the alterations.

## 5. Conclusions

We have performed single-cell transcriptome data analysis of 3389 cells from four primary glioblastoma patients and provided a detailed description of cellular heterogeneity in GBM. This study emphasized the tumor heterogeneity of GBM samples and the chromosomal aberration associated with it. We studied the cell differentiation state of glial cells, suggesting that the microglial cells are the possible target cells for GBM to invade. The filtered differentially expressed genes were used to construct the co-expression and PPI (protein-protein interaction) networks and subsequently identified the novel potential genes, which may act as therapeutic targets.

## Figures and Tables

**Table 1:** Candidate DEGs located on the chromosomes and their expression changes.

Chromosome	Gene Symbol	Start Position	End Position	Description	Expression
Chr 10	*VIM*	17228241	17237593	Vimentin	UP
	*HTRA1*	1.22 × 10^8^	1.23 × 10^8^	HtrA serine peptidase 1	DOWN
	SCD *	1 × 10^8^	1 × 10^8^	Stearoyl-CoA desaturase	DOWN
	*PSAP*	71816298	71851251	Prosaposin	DOWN
	*EGR2*	62811996	62819167	Early growth response 2	DOWN
	*PIP4K2A*	22534854	22714578	Phosphatidylinositol-5-phosphate 4-kinase type 2 α	DOWN
	*SRGN*	69057533	69104811	Serglycin	-
	*PPA1*	70202831	70233429	Inorganic pyrophosphatase 1	DOWN
	OPALIN *	96343221	96359002	Oligodendrocytic myelin paranodal and inner loop protein	NI
	PHYHIPL *	59175872	59247770	Phytanoyl-CoA 2-hydroxylase interacting protein like	DOWN
Chr 7	*ANLN*	36389806	36453791	Anillin actin binding protein	UP
	SRI *	88205118	88226993	Sorcin	UP
	*PON2*	95404863	95435329	Paraoxonase 2	UP
	*ITGB8*	20330702	20415754	Integrin subunit β 8	UP
	*PTN*	1.37 × 10^8^	1.37 × 10^8^	Pleiotrophin	UP
	*CAV1*	1.17 × 10^8^	1.17 × 10^8^	Caveolin 1	NI
	NPTX2 *	98617297	98629868	Neuronal pentraxin 2	DOWN
	*PTPRZ1*	1.22 × 10^8^	1.22 × 10^8^	Protein tyrosine phosphatase receptor type Z1	DOWN
	MEST *	1.30 × 10^8^	1.31 × 10^8^	Mesoderm specific transcript	UP
	RARRES2 *	1.50 × 10^8^	1.50 × 10^8^	Retinoic acid receptor responder 2	NI
	SEPTIN7 *	35800932	35907105	Septin 7	NI
	*CALD1*	1.35 × 10^8^	1.35 × 10^8^	Caldesmon 1	UP
	*GNAI1*	80133955	80219402	G protein subunit α I1	-
	*RAPGEF5*	22118238	22357144	Rap guanine nucleotide exchange factor 5	UP
	*EGFR*	55019021	55256620	Epidermal growth factor receptor	DOWN
	*IGFBP3*	45912245	45921874	Insulin-like growth factor binding protein 3	NI
	*COL1A2*	94394561	94431232	Collagen type I α 2 chain	DOWN
	*GPR37*	1.25E+08	1.25 × 10^8^	G protein-coupled receptor 37	NI
	*NDUFA4*	10931951	10940256	NDUFA4 mitochondrial complex associated	UP
	*GRM3*	86643914	86864884	Glutamate metabotropic receptor 3	NI
Chr 13	*TSC22D1*	44432143	44577147	TSC22 domain family member 1	-
	HSPH1 *	31134974	31162388	Heat shock protein family H (Hsp110) member 1	NI
	*COL4A2*	1.10E+08	1.11 × 10^8^	Collagen type IV α 2 chain	NI
	SLAIN1 *	77697854	77764242	SLAIN motif family member 1	DOWN
	AMER2 *	25161684	25172288	APC membrane recruitment protein 2	DOWN
	GPR183 *	99294530	99307405	G protein-coupled receptor 183	DOWN
	PCDH9 *	66302834	67230445	Protocadherin 9	DOWN
	*COL4A1*	1.10 × 10^8^	1.10 × 10^8^	Collagen type IV α 1 chain	NI
	*HMGB1*	30456704	30617597	High mobility group box 1	NI
Chr6	*TNF*	31575565	31578336	Tumor necrosis factor	UP
	F13A1 *	6144084	6320662	Coagulation factor XIII A chain	DOWN
	*MYO6*	75749203	75919537	Myosin VI	DOWN
	AKAP12 *	1.51 × 10^8^	151358559	A-kinase anchoring protein 12	DOWN
	CD109 *	73696203	73828313	CD109 molecule	UP
	*SLC16A10*	1.11 × 10^8^	111231194	Solute carrier family 16-member 10	DOWN
	UST *	1.49 × 10^8^	149076990	Uronyl 2-sulfotransferase	DOWN
	IPCEF1 *	1.54 × 10^8^	154356803	Interaction protein for cytohesin exchange factors 1	UP
	TSPYL4 *	1.16 × 10^8^	116254075	TSPY like 4	DOWN
	SELPLG *	1.09 × 10^8^	108633894	Selectin P ligand	UP
	*ENO2*	6914580	6923697	Enolase 2	NI
	*DUSP6*	89347235	89352501	Dual specificity phosphatase 6	UP
Chr 12	C3AR1 *	8056844	8066359	Complement C3a receptor 1	DOWN
	FAIM2 *	49866896	49903900	Fas apoptotic inhibitory molecule 2	UP
	*FMNL3*	49636499	49707405	Formin like 3	DOWN
	*NAV3*	77571856	78213010	Neuron navigator 3	DOWN
	*GPN3*	1.1 × 10^8^	110468721	GPN-loop GTPase 3	DOWN
	*PRPF40B*	49622717	49645129	Pre-mRNA processing factor 40 homolog B	NI
	*LGALS3*	55129252	55145430	Complement C3a receptor 1	UP
	*NDRG2*	21016763	21070872	Fas apoptotic inhibitory molecule 2	DOWN
	*HSPA2*	64535905	64543237	Formin like 3	DOWN
	*RTN1*	59595976	59871288	Neuron navigator 3	UP
	*SLC22A17*	23346304	23354991	GPN-loop GTPase 3	UP
	*PLD4*	1.05 × 10^8^	104937789	Pre-mRNA processing factor 40 homolog B	UP
Chr 17	*CCL2*	34255285	34257203	C–C motif chemokine ligand 2	UP
	*SOX9*	72121020	72126416	SRY-box transcription factor 9	DOWN
	*CCL3*	36088256	36090143	C–C motif chemokine ligand 3	UP
	*CCL4*	36103827	36105614	C–C motif chemokine ligand 4	UP
	*ABCC3*	50634881	50692253	ATP-binding cassette subfamily C member 3	UP
	*CCL4L2*	36211063	36212873	C–C motif chemokine ligand 4 like 2	UP
Chr 22	*MIF*	23894383	23895223	Macrophage migration inhibitory factor	DOWN
	*LGALS1*	37675636	37679802	Galectin 1	DOWN
	*PDGFB*	39223359	39244982	Platelet-derived growth factor subunit B	DOWN
	TNFRSF13C *	41922032	41926806	TNF receptor superfamily member 13C	DOWN
	*CECR2*	17359949	17558151	CECR2 histone acetyl-lysine reader	DOWN

* Novel genes reported in this study.

**Table 2:** Novel genes identified from our study.

Analysis Type	Novel Genes	Total
Filtered DEGs	*DHRS9, IPCEF1, TNR, MEGF11, EDIL3, PDZD2, ATP1A2, PDGFRA, LINC00632, AC243829.4, AC024909.2, MEG3, CHI3L1, FN1, IGFBP2, TNC, FCGBP, CYR61, F13A1, ANXA2, AC006064.4, ANXA1, CH25H and MIF-AS1*	24
CNV detection	*SCD, OPALIN, PHYHIPL, PSAP, SRI, NPTX2, MEST, RARRES2, SEPTIN7, HSPH1, SLAIN1, AMER2, GPR183, PCDH9, F13A1, AKAP12, CD109, UST, IPCEF1, TSPYL4, SELPLG, C3AR1, FAIM2 and TNFRSF13C*	24
Network construction	*B3GNT5, SELPLG and TPI1*	3

## Data Availability

The datasets generated during and/or analysed during the current study are available from the corresponding author upon reasonable request.

## Supplementary Materials

The following supporting information can be downloaded at: https://www.mdpi.com/article/10.3390/genes13030428/s1, Figure S1: (a) The cell quality filtering with Seurat. (b) The genes show the higher cell to cell variation (Red dots), black dots represent housekeeping genes. (c) The correlation of highly variable genes from first two PCs. (d) represent the elbow plot for the number of PCs and its standard deviation. (e) Histogram representing the number of cells in each cluster. Table S1: Particulars of the dataset obtained from GSE84465. Table S2: List of DEGs analyzed from Seurat and DESeq2. Table S3: Overlapped DEGs between Seurat, DESeq2 and highly variable genes. File S1: List of cell-type markers and variable genes. File S2: Comparison of identified potent genes in the present study with those reported in the literature.
