# Supplementary material for: Tumor Heterogeneity and Molecular Characteristics of Glioblastoma Revealed by Single-Cell RNA-Seq Data Analysis
Source: Genes (Basel). 2022 Feb 25;13(3):428. doi: 10.3390/genes13030428 (PMC8955282; doi:10.3390/genes13030428)
Supplement: Supplementary file 1 [file genes-13-00428-s001.zip › GBM-SI-Genes-23feb2022.pdf]

Supplementary information

Tumor heterogeneity and molecular characteristics of Glioblastoma revealed by single-cell RNA-seq data analysis

Dhanusha Yesudhas[1], S. Akila Parvathy Dharshini[1], Y-h. Taguchi[2] & M. Michael Gromiha[1*]

[1]Department of Biotechnology, Bhupat and Jyoti Mehta School of Biosciences, Indian Institute of Technology Madras, Chennai 600036, India

[2]Department of Physics, Chuo University, Bunkyo-ku, Tokyo 112-8551, Japan

*Corresponding author

Tel: +91 44 2257 4138

Fax: +91 44 2257 4102

E-mail: gromiha@iitm.ac.in

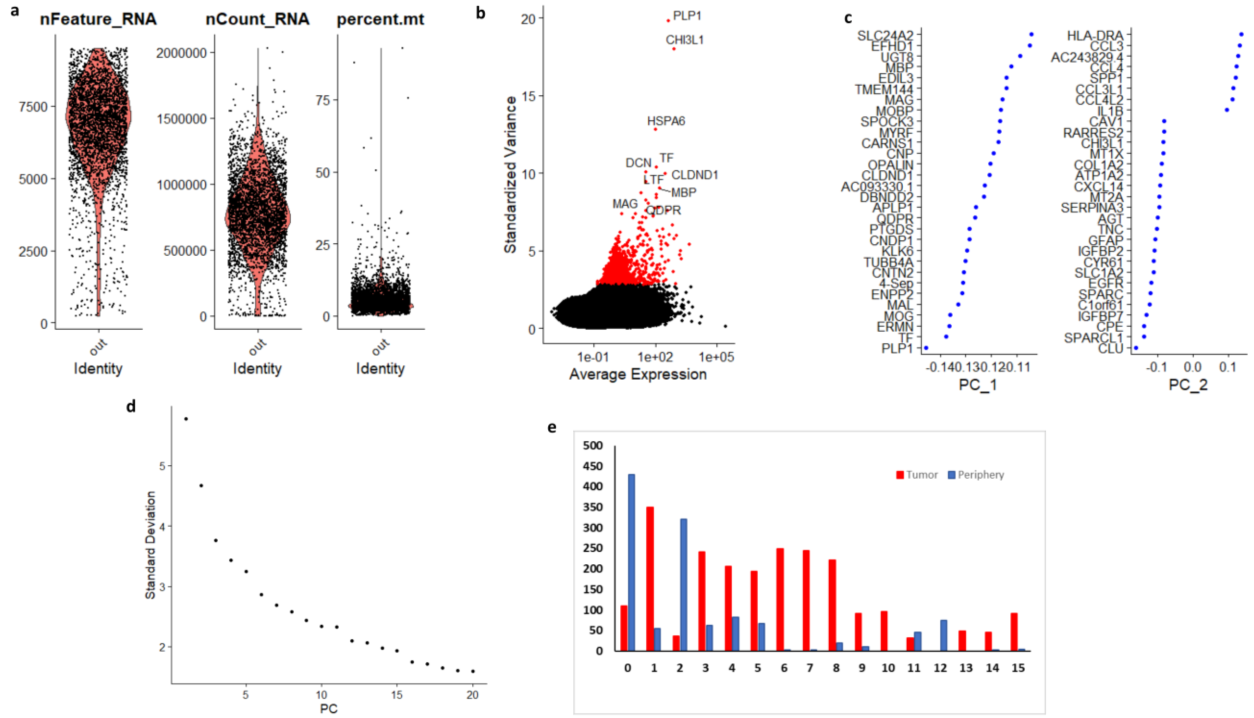

Figure S1: a) The cell quality filtering with Seurat. b) The genes show the higher cell to cell variation (Red dots), black dots represent housekeeping genes. c) The correlation of highly variable genes from first two PCs. d) represent the elbow plot for the number of PCs and its standard deviation. e) Histogram representing the number of cells in each cluster.

Table S1: Particulars of the dataset obtained from GSE84465

sample	age	grade	No.of cells-Tumor core	No.of cells-Periphery core	Total
BT_S1	55	GBM (IV)	433	56	489
BT_S2	54	GBM (IV)	723	446	1169
BT_S4	60	GBM (IV)	980	562	1542
BT_S6	48	GBM (IV)	207	182	389

Table S2: List of DEGs analyzed from Seurat and DESeq2.

Gene	baseMean	log2FoldChange	lfcSE	stat	pvalue	padj
DHRS9	48.82053	-5.29234	0.288533	-14.8764	4.69E-50	2.12E-47
IPCEF1	53.10857	-4.11055	0.21704	-14.3317	1.39E-46	5.56E-44
TNR	183.4263	-3.93974	0.343757	-8.55181	1.21E-17	1.29E-15
MEGF11	52.43762	-3.93523	0.360671	-8.13823	4.01E-16	3.86E-14
EDIL3	48.96293	-3.47214	0.407837	-6.06159	1.35E-09	6.23E-08
PDZD2	52.57034	-3.41071	0.145358	-16.5847	8.99E-62	6.37E-59
ATP1A2	90.05974	-3.19878	0.176803	-12.4363	1.66E-35	4.15E-33
PDGFRA	58.78193	-3.06634	0.30484	-6.77843	1.21E-11	7.48E-10
TNF	75.88377	-3.01342	0.340066	-5.92069	3.21E-09	1.39E-07
SPOCK1	102.3572	-3.00894	0.344898	-5.82473	5.72E-09	2.42E-07
CX3CR1	96.157	-3.00793	0.270107	-7.43383	1.05E-13	8.01E-12
SLC6A1	52.74271	-2.97511	0.123666	-15.9712	2.03E-57	1.27E-54
PLD4	68.89382	-2.97135	0.256422	-7.6879	1.50E-14	1.22E-12
NLGN3	96.34893	-2.89169	0.263393	-7.18202	6.87E-13	4.78E-11
SULF2	63.37779	-2.77187	0.231434	-7.65606	1.92E-14	1.53E-12
P2RY12	53.83475	-2.74123	0.271098	-6.42286	1.34E-10	7.12E-09
LINC00632	118.537	-2.73523	0.100501	-17.2658	8.51E-67	7.54E-64
OLIG1	85.30542	-2.73416	0.265278	-6.53712	6.27E-11	3.50E-09
OMG	61.85165	-2.73391	0.35442	-4.89223	9.97E-07	3.03E-05
FMNL3	112.2576	-2.68436	0.172437	-9.768	1.54E-22	2.30E-20
EGR3	68.76833	-2.66892	0.205202	-8.13306	4.19E-16	4.01E-14
IL1B	328.3913	-2.64809	0.26453	-6.23027	4.66E-10	2.3E-08
C3AR1	136.4042	-2.61296	0.209024	-7.71662	1.19E-14	9.88E-13
AC243829.4	912.5554	-2.60257	0.184951	-8.66483	4.52E-18	4.95E-16
CCL3	2095.761	-2.56859	0.208475	-7.5241	5.31E-14	4.12E-12
DNER	129.6054	-2.53655	0.267093	-5.75286	8.77E-09	3.59E-07
CCL3L1	957.6199	-2.53056	0.228902	-6.68653	2.29E-11	1.33E-09
SELPLG	97.50134	-2.52662	0.229384	-6.65528	2.83E-11	1.63E-09
CCL4	1783.144	-2.46165	0.2334	-6.26242	3.79E-10	1.89E-08
ST6GAL1	139.5148	-2.44215	0.191302	-7.53859	4.75E-14	3.70E-12
SLC1A2	184.5836	-2.4357	0.117122	-12.2582	1.52E-34	3.67E-32
SLC22A17	96.20043	-2.38559	0.229074	-6.04868	1.46E-09	6.7E-08
B3GNT5	99.28527	-2.37037	0.162585	-8.42862	3.50E-17	3.57E-15
EGR2	238.7823	-2.34813	0.200004	-6.74054	1.58E-11	9.39E-10
GPR37L1	86.75378	-2.34124	0.150785	-8.89505	5.84E-19	7.01E-17
HTRA1	350.9456	-2.33465	0.140195	-9.51997	1.73E-21	2.49E-19
CCL2	281.1884	-2.33084	0.257685	-5.1646	2.41E-07	8.09E-06
CH25H	113.0968	-2.31614	0.304562	-4.32143	1.55E-05	0.000395
SPARCL1	327.338	-2.26348	0.21886	-5.77297	7.79E-09	3.21E-07
MTSS1	111.8151	-2.23598	0.133795	-9.23785	2.51E-20	3.36E-18
AC024909.2	129.3276	-2.15614	0.188432	-6.13557	8.49E-10	4.02E-08
CCL4L2	1167.826	-2.1321	0.246026	-4.60154	4.19E-06	0.000116
MEG3	168.6652	-2.10667	0.166323	-6.65377	2.86E-11	1.64E-09
DUSP6	397.7049	-2.09053	0.153777	-7.09166	1.33E-12	8.94E-11
CNP	182.7913	-2.07893	0.129232	-8.34877	6.90E-17	6.75E-15
SCD5	163.4324	-2.07233	0.113607	-9.43891	3.77E-21	5.34E-19

NCAM1	91.18541	-2.01595	0.231474	-4.38903	1.14E-05	0.000297
BTG2	164.5133	-1.9547	0.173681	-5.49685	3.87E-08	1.44E-06
ALDOC	326.5332	-1.94665	0.217854	-4.34532	1.39E-05	0.000357
APOD	415.1671	-1.93026	0.269011	-3.45807	0.000544	0.010177
PLLP	191.7838	-1.81409	0.113073	-7.19962	6.04E-13	4.23E-11
NFKBID	159.3479	-1.72074	0.168438	-4.27897	1.88E-05	0.00047
CSF1R	173.6709	-1.70807	0.161933	-4.37261	1.23E-05	0.000318
TSPAN7	189.1584	-1.69589	0.227495	-3.05891	0.002221	0.034941
CD83	460.4818	-1.68964	0.169855	-4.06017	4.90E-05	0.001129
TSC22D1	204.3057	-1.68924	0.198265	-3.47636	0.000508	0.009558
A2M	616.6864	-1.67193	0.166939	-4.02503	5.70E-05	0.001299
GPRC5B	179.9042	-1.67011	0.210087	-3.1897	0.001424	0.023644
TRIB1	172.4281	-1.66746	0.184292	-3.62176	0.000293	0.005811
NR4A1	261.8586	-1.63761	0.124766	-5.11041	3.21E-07	1.06E-05
GSN	275.941	-1.51055	0.108254	-4.71627	2.40E-06	6.88E-05
FTL	5827.682	1.315785	0.077336	4.083305	4.44E-05	0.001028
TPI1	546.8562	1.403672	0.097382	4.145247	3.39E-05	0.000807
IFI30	319.3334	1.4409	0.145324	3.033903	0.002414	0.037776
GAPDH	2410.082	1.49742	0.071354	6.971166	3.14E-12	2.07E-10
RPLP1	377.8319	1.506001	0.064164	7.886081	3.12E-15	2.76E-13
AC006064.4	651.7123	1.579643	0.06633	8.738798	2.36E-18	2.64E-16
PTN	169.528	1.594212	0.192007	3.094734	0.00197	0.031545
NAMPT	244.3025	2.081227	0.115699	9.345168	9.17E-21	1.26E-18
TMSB10	302.206	2.088534	0.080737	13.48241	1.99E-41	6.34E-39
MIF	169.375	2.106475	0.113137	9.779919	1.37E-22	2.07E-20
MIF-AS1	124.1214	2.136963	0.110949	10.24762	1.21E-24	1.97E-22
PLTP	136.8626	2.184172	0.137561	8.608315	7.41E-18	7.96E-16
LDHA	472.8694	2.508327	0.114734	13.14625	1.79E-39	5.14E-37
IGFBP7	102.3825	2.628963	0.213996	7.612104	2.70E-14	2.15E-12
LGALS1	180.5283	2.750834	0.125023	14.00404	1.47E-44	5.30E-42
ANXA1	177.666	2.770735	0.174805	10.12977	4.08E-24	6.42E-22
MT2A	407.2491	2.818871	0.132495	13.72781	6.92E-43	2.33E-40
VIM	610.6427	2.832405	0.125698	14.57787	3.88E-48	1.65E-45
TIMP1	154.3545	2.868781	0.157607	11.85725	1.97E-32	4.51E-30
THBS1	187.4487	2.913584	0.228692	8.367533	5.88E-17	5.84E-15
S100A6	99.27232	3.093783	0.13386	15.64161	3.79E-55	2.07E-52
TGFBI	170.9226	3.178653	0.211241	10.31361	6.12E-25	1.02E-22
CD44	164.8997	3.202227	0.156787	14.04595	8.16E-45	2.99E-42
ANXA2	245.9797	3.372478	0.153835	15.42227	1.16E-53	6.01E-51
GFAP	649.5314	3.37664	0.15114	15.72476	1.02E-55	5.88E-53
F13A1	63.06351	3.513745	0.260556	9.647635	5.03E-22	7.32E-20
CYR61	92.81008	3.55395	0.30466	8.382959	5.16E-17	5.15E-15
S100A10	132.493	3.736323	0.154551	17.70497	3.84E-70	4.08E-67
FCGBP	258.404	4.544667	0.253759	13.96862	2.42E-44	8.44E-42
TNC	74.03079	4.850649	0.220427	17.46901	2.47E-68	2.38E-65
IGFBP2	73.36874	4.85922	0.159827	24.14625	8.18E-129	3.47E-125
FN1	117.2704	4.877895	0.173356	22.3695	7.80E-111	2.76E-107
CHI3L1	517.2369	5.97367	0.29588	16.80974	2.07E-63	1.57E-60

Table S3: Overlapped DEGs between Seurat, DESeq2 and highly variable genes.

Gene	baseMean	log2FoldChange	lfcSE	stat	pvalue	padj
TIMP1	154.3545	2.868781	0.157607	11.85725	1.97E-32	4.51E-30
CCL2	281.1884	-2.33084	0.257685	-5.1646	2.41E-07	8.09E-06
SLC1A2	184.5836	-2.4357	0.117122	-12.2582	1.52E-34	3.67E-32
FN1	117.2704	4.877895	0.173356	22.3695	7.80E-111	2.76E-107
IGFBP2	73.36874	4.85922	0.159827	24.14625	8.18E-129	3.47E-125
TNR	183.4263	-3.93974	0.343757	-8.55181	1.21E-17	1.29E-15
MT2A	407.2491	2.818871	0.132495	13.72781	6.92E-43	2.33E-40
IL1B	328.3913	-2.64809	0.26453	-6.23027	4.66E-10	2.3E-08
GFAP	649.5314	3.37664	0.15114	15.72476	1.02E-55	5.88E-53
CHI3L1	517.2369	5.97367	0.29588	16.80974	2.07E-63	1.57E-60
THBS1	187.4487	2.913584	0.228692	8.367533	5.88E-17	5.84E-15
CYR61	92.81008	3.55395	0.30466	8.382959	5.16E-17	5.15E-15
SPARCL1	327.338	-2.26348	0.21886	-5.77297	7.79E-09	3.21E-07
IGFBP7	102.3825	2.628963	0.213996	7.612104	2.70E-14	2.15E-12
CNP	182.7913	-2.07893	0.129232	-8.34877	6.90E-17	6.75E-15
APOD	415.1671	-1.93026	0.269011	-3.45807	0.000544	0.010177
TNF	75.88377	-3.01342	0.340066	-5.92069	3.21E-09	1.39E-07
CCL4	1783.144	-2.46165	0.2334	-6.26242	3.79E-10	1.89E-08
FCGBP	258.404	4.544667	0.253759	13.96862	2.42E-44	8.44E-42
CCL4L2	1167.826	-2.1321	0.246026	-4.60154	4.19E-06	0.000116
CCL3L1	957.6199	-2.53056	0.228902	-6.68653	2.29E-11	1.33E-09
AC243829.4	912.5554	-2.60257	0.184951	-8.66483	4.52E-18	4.95E-16
CCL3	2095.761	-2.56859	0.208475	-7.5241	5.31E-14	4.12E-12

Supplementary File S1: List of cell-type markers and variable genes

Supplementary File S2: Comparison of identified potent genes in the present study with those reported in the literature
